# Peli1 negatively regulates type I interferon induction and antiviral immunity in the CNS

**DOI:** 10.1186/s13578-015-0024-z

**Published:** 2015-06-24

**Authors:** Yichuan Xiao, Jin Jin, Qiang Zou, Hongbo Hu, Xuhong Cheng, Shao-Cong Sun

**Affiliations:** Department of Immunology, The University of Texas MD Anderson Cancer Center, 7455 Fannin Street, Box 902, Houston, TX 77030 USA; Institute of Health Sciences, Shanghai Institutes for Biological Sciences, Chinese Academy of Science/Shanghai Jiao Tong University School of Medicine, Shanghai, 200031 China; The University of Texas Graduate School of Biomedical Sciences at Houston, Houston, TX 77030 USA

**Keywords:** Peli1, Type I interferon, Antiviral immunity, CNS

## Abstract

**Background:**

Type I interferons (IFN-Is) serve as mediators of antiviral innate immunity and also regulate adaptive immune responses. The molecular mechanism that regulates virus-induced IFN-I production, particularly in tissue-resident immune cells, is incompletely understood.

**Results:**

Here we identified the E3 ubiquitin ligase Peli1 as a negative regulator of IFN-I induction in microglia, innate immune cells of the central nervous system (CNS). Peli1 deficiency profoundly promoted IFN-β expression in microglia in response to *in vitro* stimulation by toll-like receptor (TLR) ligands or a CNS-tropic virus, the vascular stomatitis virus (VSV). Upon intranasal infection with VSV, the Peli1-deficient mice displayed heightened *in vivo* IFN-I responses in the CNS, coupled with reduced brain viral titer and increased survival rate.

**Conclusions:**

These results establish Peli1 as an innate immune regulator in the CNS that modulates the threshold of IFN-I responses against viral infections.

## Background

Type I interferons (IFN-Is) provide one of the body’s primary defense systems against viral infections. The high susceptibility of type I IFN receptor (IFNAR)-deficient mice to infection by a variety of viruses provides strong evidence for the major role of the IFN system in anti-viral innate immunity [1]. In addition, IFN-Is also play an important role in modulating the development of adaptive immune responses [2]. Induction of IFN-Is is mediated by pattern-recognition receptors (PRRs), such as the toll-like receptors (TLRs) and the cytoplasmic RIG-I-like receptors (RLRs). The PRRs can efficiently detect viral infections through recognition of molecular patterns associated with viral genomes and replication products [3–5]. The TLR family is composed of members that signal through two major adaptors, MyD88 and TRIF. The TRIF-dependent TLRs, TLR3 and TLR4, as well as the RLRs stimulate *Ifnb* gene expression via activation of two homologous kinases, TBK1 and IKKε. These kinases in turn phosphorylate the transcription factor IRF-3, which causes IRF-3 dimerization and translocation to the nucleus, where it mediates the transactivation of the *Ifnb* gene [6, 7].

Although IFN-I induction has been extensively studied using macrophages and dendritic cells, IFN-Is are also produced by tissue resident cells and play a tissue-specific role in mediating antiviral innate immunity and regulating immune homeostasis [2, 8]. In particular, the microglia are resident macrophages of the central nervous system (CNS) that are important for immune responses against infections in the CNS [9, 10]. These cells, which seed the parenchyma of the CNS early during embryonic development, express various PRRs, including the TLRs and RLRs, and become rapidly activated upon detection of infections and tissue damage [11–13]. In addition to mediating antiviral innate immunity, IFN-Is play an important role in regulating other types of immune responses, thereby exerting pathogenic or protective roles in the CNS [13]. Thus, the production of IFN-Is is subject to both positive and negative mechanisms of regulation.

Despite the extensive studies on the core components of the IFN-I induction pathway, the regulatory factors are still poorly defined. We have recently shown that the E3 ubiquitin ligase Peli1 (also called Pellino1) is required for TLR-stimulated proinflammatory cytokine expression in microglia, although its role in regulating IFN-I expression has remained unclear [14]. Unlike peripheral macrophages, which express both Peli1 and its homologs, Peli2 and Peli3, microglia predominantly express Peli1 [14]. We found in the present study that in contrast to its positive role in regulating proinflammatory cytokine induction, Peli1 is a potent negative regulator of IFN-I induction by TLR ligands and viruses. In response to intranasal infection by a neurotropic virus, vascular stomatitis virus (VSV), the Peli1-deficient mice expressed elevated levels of IFNα and IFN-β in the microglia of CNS, coupled with reduced brain viral titer and increased rate of mouse survival. These findings establish Peli1 as a negative regulator of IFN-I induction and antiviral innate immunity in the CNS.

## Results

### Peli1 negatively regulates TLR-mediated IFN-β induction in microglia

**Fig. 1:**
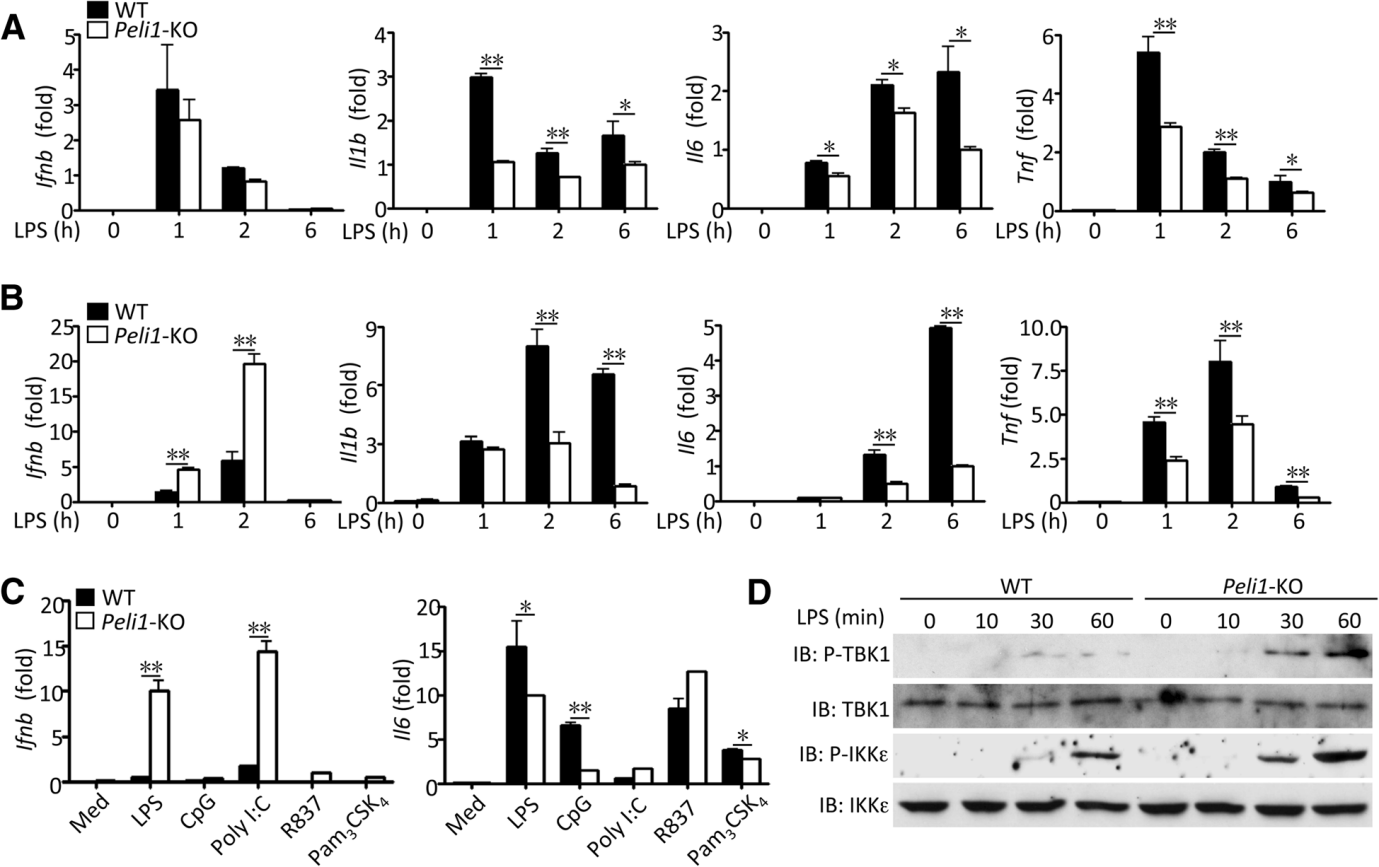
*Peli1* negatively regulates TLR-mediated IFN-β induction in microglia. **a-c** QPCR analysis of relative mRNA expression for the indicated genes in WT and *Peli1*-KO bone marrow-derived macrophages (BMDM) (**a**) or microglia (**b,c**) stimulated with the indicated ligands for different TLRs: TLR4 (LPS, 100 ng/ml), TLR9 (CpG, 2.5 μM), TLR7/8 (R837, 1 μg/ml), TLR1/2 (Pam_3_CSK_4_, 1 μg/ml), and TLR3 (poly(I:C), 10 μg/ml). *P < 0.05 and **P < 0.01. **d** IB analysis of phosphorylated (P-) and total TBK1 and IKKε in whole-cell lysates of WT or *Peli1*-KO microglia stimulated with LPS (100 ng/ml)

To investigate the role of Peli1 in regulating IFN-I induction, we prepared primary BMDM and microglia from *Peli1*-KO mice and wildtype (WT) control mice. In contrast to the BMDMs expressing an E3 ligase-defective Peli1 mutant [15], the *Peli1*-KO BMDMs did not show significant reduction in LPS-stimulated IFN-β induction (Fig. 1a). On the other hand, the Peli1 deficiency reduced the induction of several proinflammatory cytokine genes, likely due to the role of Peli1 in mediating TRIF-dependent NF-κB activation [16]. Since BMDMs express different Peli family members at comparably levels [17], Peli1 might have functional redundancy with the other members. We thus examined the role of Peli1 in IFN-β regulation in microglia, in which Peli1 represents the predominant Peli family member [14]. As we previously reported [14], the Peli1 deficiency in microglia profoundly inhibited the induction of several proinflammatory cytokines by LPS and other TLR ligands (Fig. 1b, c). To our surprise, the loss of Peli1 in microglia resulted a striking upregulation of IFN-β induction stimulated by LPS as well as by several other TLR ligands (Fig. 1b, c). These results suggest that Peli1 functions as a negative regulator of IFN-β induction in microglia.

To delineate the molecular mechanism by which Peli1 negatively regulates IFN-β induction, we examined the effect of Peli1 deficiency on the activation of two major IFN-stimulating kinases, TBK1 and IKKε, based on their phosphorylation. Stimulation of WT microglia by LPS led to the phosphorylation of both TBK1 and IKKε (Fig. 1d). Moreover, the Peli1 deficiency substantially enhanced the LPS-stimulated TBK1/IKKε phosphorylation. These results suggest that Peli1 negatively regulates TLR-mediated IFN-I induction through inhibition of signaling events involved in the activation of TBK1 and IKKε.

### Peli1 deficiency promotes IFN-β production in brain upon LPS stimulation

**Fig. 2:**
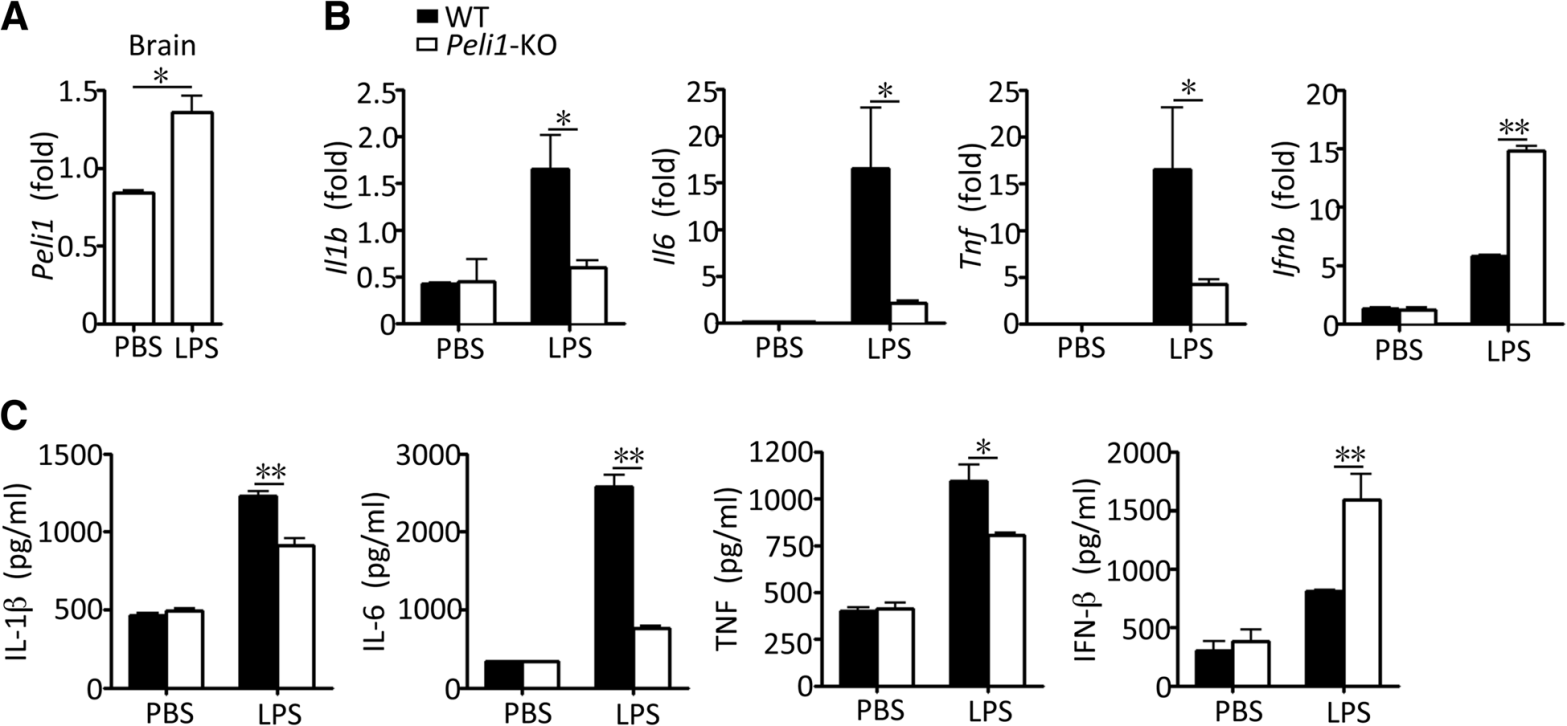
Peli1 deficiency promotes IFN-β production in brain upon LPS stimulation. **a-c** QPCR analysis of relative mRNA expression for Peli1 and the indicated genes in the brains of WT and *Peli1*-KO mice that were stereotaxically injected with PBS or LPS. **c** ELISA of the indicated cytokines in the brain homogenate of WT and *Peli1*-KO mice that were stereotaxically injected with PBS or LPS. *P < 0.05; **P <0.01

To examine the *in vivo* role of Peli1 in regulating IFN-I induction, we stereotaxically injected LPS into cerebrospinal fluid (CSF) and examined the IFN-I induction in the mouse brain. We found that injection of LPS stimulated the expression of Peli1 in the brain (Fig. 2a). Consistent with our previous work [14], the LPS-stimulated induction of several proinflammatory cytokines was significantly decreased in the brain of *Peli1*-KO mice, as compared to that of the WT mice. In contrast, the Peli1 deficiency substantially enhanced the *Ifnb* gene induction following the LPS injection (Fig. 2b). Similarly, ELISA also revealed an elevated level of IFN-β protein in the whole brain homogenization of the LPS-injected Peli1-deficient mice (Fig. 2c). These results are consistent with the *in vitro* studies using the Peli1-deficient microglia (Fig. 1b) and further establish an *in vivo* role for Peli1 in negatively regulating IFN-β production in the brain.

### Peli1 negatively regulates VSV-mediated IFN-I expression

**Fig. 3:**
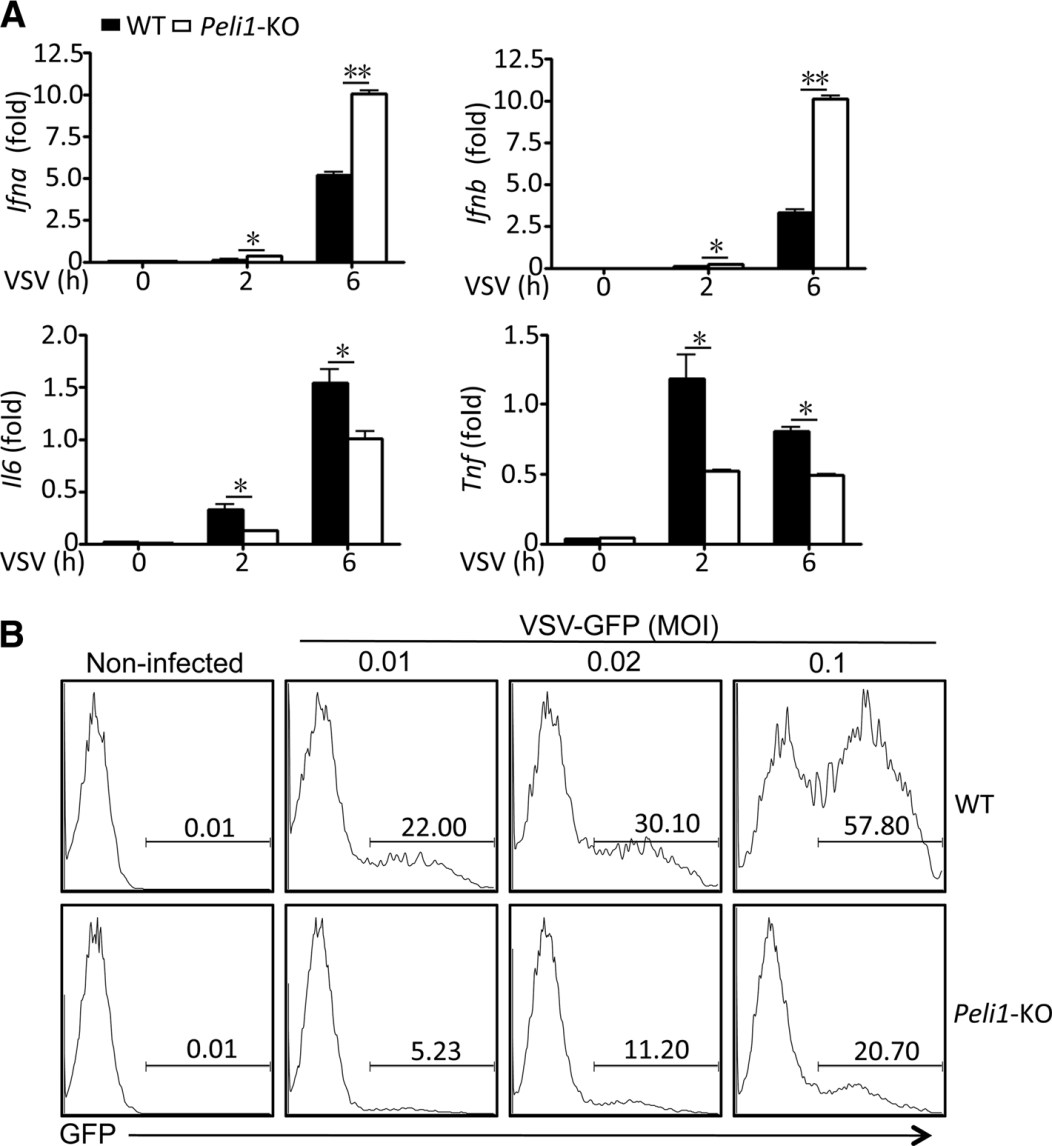
Peli1 negatively regulate VSV-mediated IFN-I induction. **a** QPCR analysis of the indicated genes in WT and *Peli1*-KO microglia infected with VSV *in vitro*. **b** Flow cytometry analysis of the percentage of VSV-GFP-infected WT and *Peli1*-KO microglia. Numbers in the panels represent the percentage of GFP^+^ (VSV-infected) microglia. *P < 0.05; **P <0.01

Microglia serve as a major type of CNS-resident cells that respond to viral infections [18–20]. To investigate the role of Peli1 in regulating antiviral immune responses in microglia, we employed VSV, a virus known to infect microglia and CNS [21, 22]. Infection of microglia with VSV led to the induction of both IFN-Is and proinflammatory cytokines. Furthermore, Peli1 deficiency in microglia significantly enhanced the induction of *Ifna* and *Ifnb* genes and concomitantly inhibited the induction of the *Il6* and *Tnf* genes (Fig. 3a). To examine the functional role of Peli1 in mediating anti-VSV host defense, we infected the Peli1-deficient and WT microglia with VSV-GFP, which allows quantification of the infected cells by flow cytometry based GFP expression. Consistent with their hyper-production of IFN-Is, the *Peli1*-KO microglia were substantially more resistant to VSV infection, as evidenced by the much lower level of VSV-infected cells (Fig. 3b). These results suggest that Peli1 negatively regulate anti-VSV immune responses in microglia.

### Peli1 deficiency promotes host defense against viral infection in CNS

**Fig. 4:**
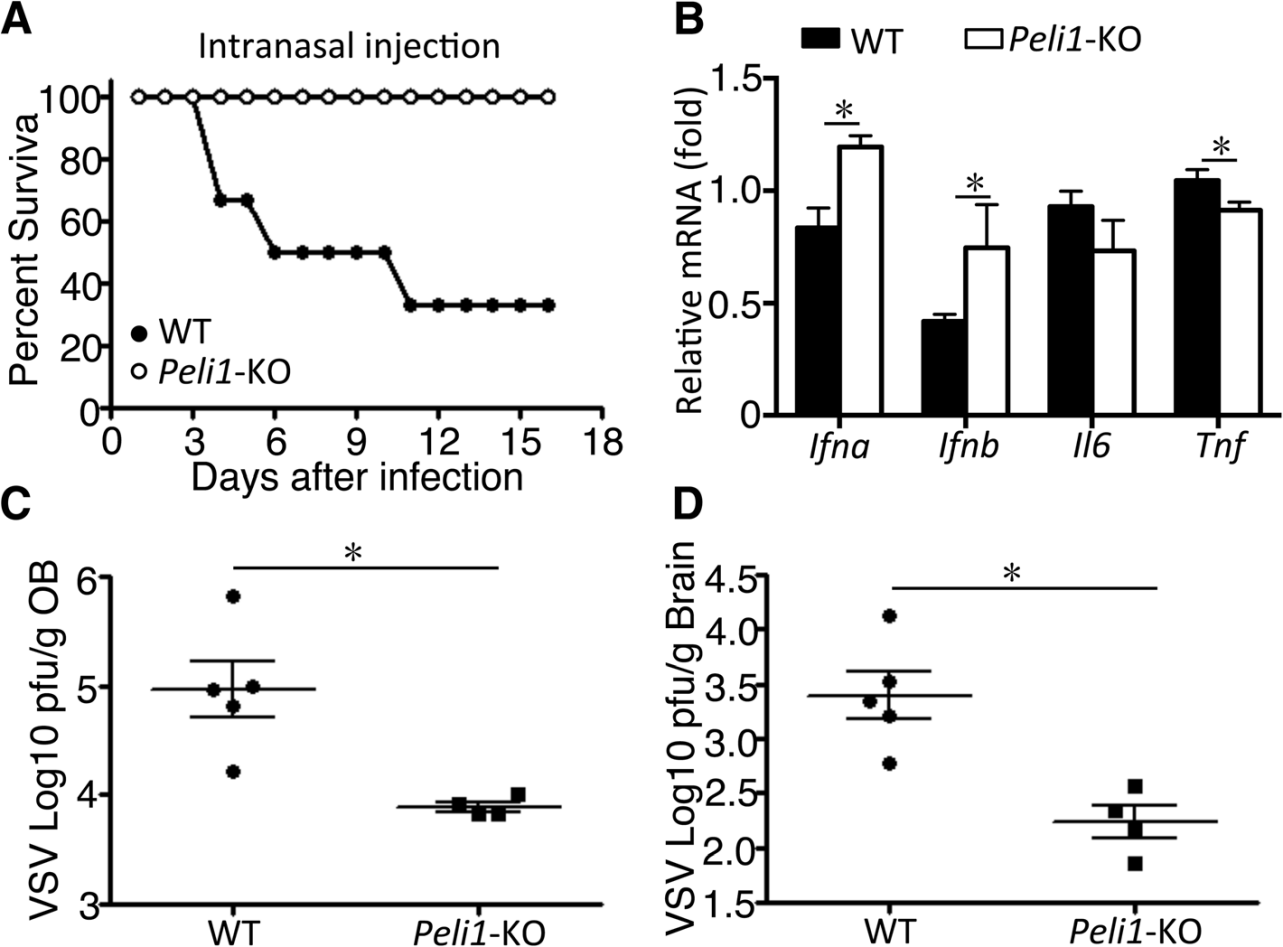
Peli1 deficiency promotes host defense against viral infection in the CNS. **a** Survival of WT (*n* = 7) and *Peli1*-KO (*n* = 6) mice after intranasal infection with 1 × 10^7^ pfu of VSV. **b** QPCR analysis of the indicated genes in the brain of WT and *Peli1*-KO mice 24 h after intranasal infection with 1 × 10^7^ pfu of VSV. **c, d** VSV titer of the OB (**c**) and brain (**d**) in WT and *Peli1*-KO mice on day two after VSV intranasal infection, plotted as pfu/g tissue with mean on log scale

To examine the *in vivo* role of Peli1 in regulating antiviral function, we challenged the *Peli1*-KO and WT control mice with VSV through intranasal injection, a route that is known to lead to CNS infection [21, 23]. Compared with the WT control mice, the *Peli1*-KO mice were remarkably more resistant to VSV infection, as revealed by the much improved survival rate (Fig. 4a). This phenotype of the *Peli1*-KO mice was associated with significantly enhanced expression of IFN-Is in the brain (Fig. 4b), and remarkably lower VSV titer in the homogenates of the olfactory bulb (OB) and brain (Fig. 4c, d).

**Fig. 5:**
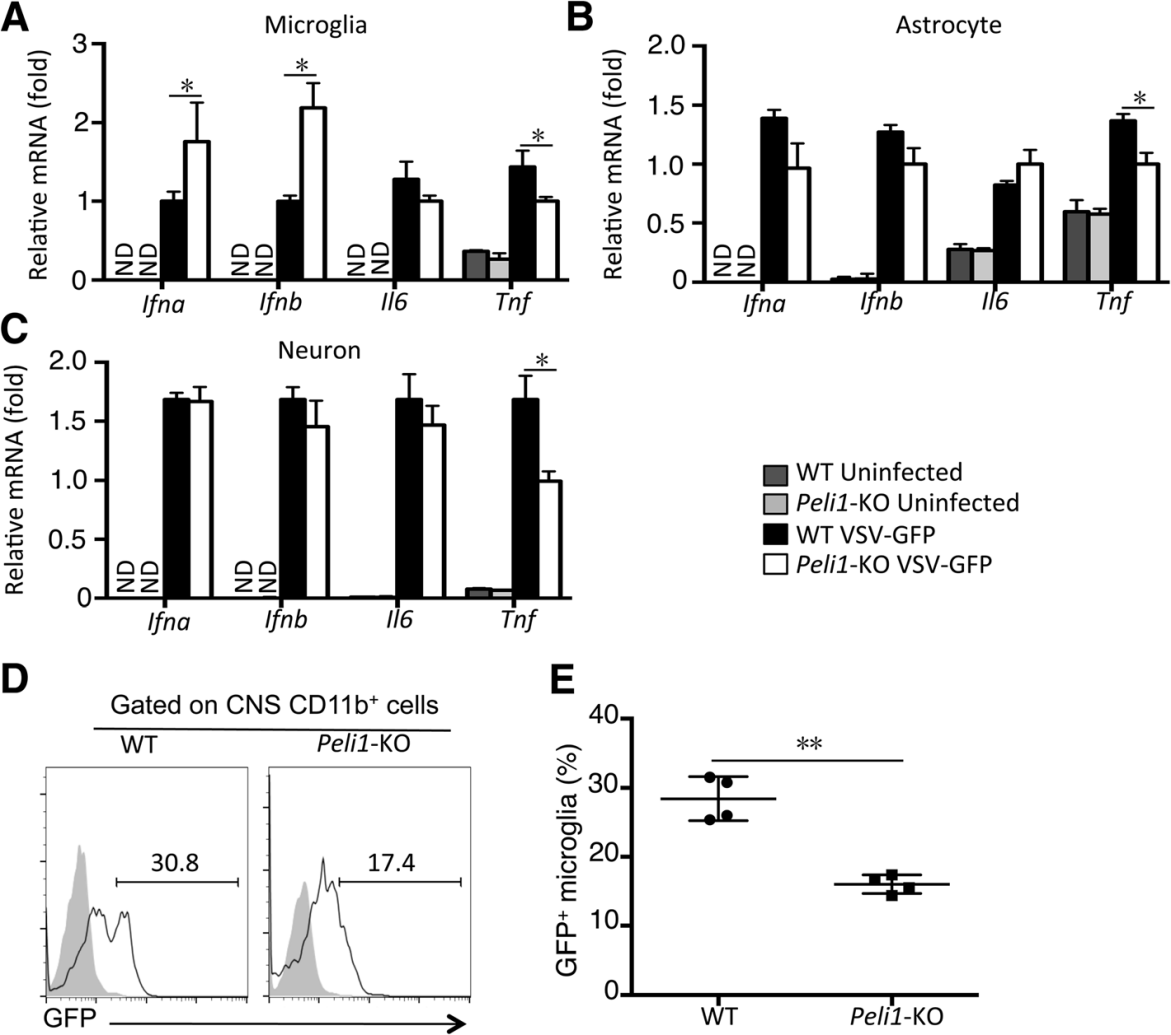
Peli1 deficiency promotes IFN-I expression and anti-viral defense by microglia in an *in vivo* model of VSV infection. **a-c** QPCR analysis of the indicated genes in FACS-sorted microglia, astrocytes, and neuron derived from the WT or *Peli1*-KO mice 24 h after intracranial infection with VSV-GFP (1 × 10^7^ pfu). **d,e** Flow cytometric analysis of GFP expression in CD11b^+^ microglia isolated from the brain of WT or *Peli1*-KO mice 24 h after intracranial infection with VSV-GFP (1 × 10^7^ pfu). Data are presented as a representative plot (**d**) and summary graph of the percentage of GFP^+^ microglia (**b**). *P < 0.05; **P < 0.01

To identify the neural cell linage that is responsible for the production of IFN-Is against virus infection, we infected WT and *Peli1*-KO mice with VSV-GFP through intracranial injection, and then isolated different type of neural cells by FACS sorting for QPCR analysis. The microglia isolated from VSV-GFP-infected *Peli1*-KO mice expressed a significantly higher level of *Ifna* and *Ifnb* than the microglia derived from the VSV-GFP-infected WT mice (Fig. 5a). In contrast, neither astrocytes nor neurons, isolated from the VSV-GFP-infected WT and *Peli1*-KO mice, had significant differences in IFN-I expression (Fig. 5b, c). We also found that the *Peli1*-KO microglia exhibited significantly reduced GFP expression compared to the WT microglia, suggesting augmented anti-VSV function of the *Peli1*-KO microglia (Fig. 5d, e). This result was consistent with that obtained with the *in vitro* microglial infection model (Fig. 3b). Taken together, these data suggest that Peli1 negatively regulates CNS host defense against VSV infection through suppressing IFN-I production in microglia.

## Discussion

In the present study, we identified the E3 ubiquitin ligase Peli1 as a pivotal negative regulator of IFN-I induction in microglia of the CNS. The Peli1 deficiency profoundly promoted the induction of IFN-Is in microglia in response to stimulation by the ligands of TRIF-dependent TLRs, poly(I:C) and LPS, or infection by the RNA virus VSV. The hyper-production of IFN-Is by the Peli1-deficient microglia was associated with enhanced protection against VSV infection. The *Peli1*-KO mice were also more resistant to intranasal infection with VSV, coupled with heightened production of IFN-Is in the CNS.

The microglia-specific role of Peli1 in negatively regulating IFN-I expression is at least partially due to its cell-specific expression. We have previously shown that Peli1 deficiency has no appreciable effect on the induction of IFN-Is in peripheral innate immune cells and MEFs [16]. Interestingly, however, a Peli1 knockin study demonstrates that IFN-β induction is attenuated in myeloid cells and MEFs expressing a Peli1 mutant lacking E3 ligase activity [15]. It is currently unclear whether this discrepancy is due to cell-type specific functions of Peli1 or the differences between expression of a Peli1 mutant and complete ablation of Peli1. One possibility is that the E3-defective Peli1 may exert an active role by binding to, and modulate the function of, other Peli family members. In this regard, Peli3 serves as a negative regulator of IFN-I induction by TLR3 and viruses [24]. Future studies will examine whether the mutant form of Peli1 is able to bind Peli3 and promote the negative role of Peli3 in IFN-I regulation. Notwithstanding, our present study identified Peli1 as a negative regulator of IFN-I induction in microglia in response to TLR stimulation and VSV infection. In these CNS resident macrophages, Peli1 is the predominant Peli family member [14], which allows us to investigate Peli1 function without a major influence from other Peli family members. Our findings suggest that Peli1 and Peli3 share similar functions in the regulation of IFN-I induction, which is consistent with the high level of structural homology between these two Peli family members.

Although how precisely Peli1 negatively regulates IFN-I induction in microglia remains to be further investigated, our present study suggests that Peli1 controls a signaling step that mediates activation of the protein kinases TBK1 and IKKε. Peli1-deficient microglia were hyperresponsive to LPS stimulation in the activation of both TBK1 and IKKε. Previous studies have identified TRAF3 a central mediator of IFN-I induction by both TLR ligands and viruses [25, 26]. Notably, we have previously shown that the Peli1 deficiency causes accumulation of TRAF3 in microglia following TLR stimulation [14]. It is thus possible that the accumulated TRAF3 expression in Peli1-deficient microglia may contribute to the enhanced activation of TBK1 and IKKε and hyper-induction of IFN-Is. This possibility will be examined in future studies.

## Conclusions

The findings of the present study establish Peli1 as a negative regulator of IFN-I induction in microglia and an important modulator of antiviral immune responses in the CNS.

## Methods

### Mice

*Peli1*-deficient mice (on the C57BL/6 × 129/Sv background) have been described. *Peli1*^+/−^ heterozygous mice were bred to generate age-matched *Peli1*^−/−^ (*Peli1*-KO) and *Peli1*^+/+^ (WT) mice. Green fluorescence protein (GFP) transgenic (C57BL/6-Tg(CAG-EGFP)10sb/J) mice were from The Jackson Laboratory. Mice were maintained in a specific pathogen-free facility, and all animal experiments were in accordance with protocols approved by the Institutional Animal Care and Use Committee of the University of Texas MD Anderson Cancer Center.

### Antibodies and reagents

Antibodies for TBK1 and phospho-TBK1 were purchased from Cell Signaling Technology Inc. The anti-IKKε and anti-phospho-IKKε antibodies were from Santa Cruz and Millipore, respectively. LPS (derived from *Escherichia coli* strain 0127:B8) was from Sigma-Aldrich, poly(I:C) was from Amersham, CpG (1668) was from TIB Molbio, Pam_3_CSK_4_ was from Alexis Biochemicals, and R-837 was from Invivogen.

### Stereotaxic injection

We slowly injected PBS, LPS or VSV-GFP into the cerebrospinal fluid (CSF) (AP, −0.4 mm; ML, −0.8 mm; and DV, −2.5 mm) of *Peli1*-KO or wild-type control mice as described previously [14].

### BM chimeras

WT and *Peli1*-KO mice (8-weeks-old) were lethally irradiated (950 rads) and reconstituted with mononuclear BM cells (1 × 10^7^) from the GFP transgenic mice. After 8 weeks, the chimeric mice were immunized for EAE induction as described previously [14].

### Flow cytometry

Cell suspensions were subjected to flow cytometry analyses as previously described using a LSRII flow cytometer [14].

### Viruses and infections

VSV (Indiana strain) and VSV expressing GFP (VSV-GFP) were provided by Glen Barber. For infections, 1 × 10^7^ pfu of VSV in 30 μl of endotoxin-free PBS was administered in drops (~5 ml/drop) to isofluorane-anesthetized 8–12 week-old mice, with PBS-only as control. Thereafter, mice were monitored daily for disease symptoms and mortality. In some experiment, mice were sacrificed 24 h after virus infection, the brain were collected for RNA extraction or for preparation of neural cells by using Neural Tissue Dissociation Kit (Miltenyi Biotech). The isolated neural cells were subjected for flow cytometric analysis or for FACS sorting of microglia (CD11b^+^), astrocyte (GLAST-1^+^), and neuron (CD11b^−^CD31^−^GLAST1^−^O4^−^) by using the Neuron isolation kit from Miltenyi Biotec.

### Virus quantification

For quantification of infectious VSV in organs, mice were anesthetized with pentobarbital (150 mg/kg). Organs were snap-frozen in liquid nitrogen, weighed, pestle/tube-homogenized in 1 ml of PBS per brain or lung or 0.1 ml per pair of olfactory bulbs, and virus was titered in 10-fold serial dilutions on BHK21 cells by plaque assay. Results are expressed as plaque-forming units (pfu) per gram of tissue.

### Primary microglia culture

Mixed glial cultures were prepared from 1- to 2-day-old mice. In brief, after removing the meninges, brains were dissociated by 0.25 % trypsin, and were filtered with a 40-μm mesh. Cells were cultured in DMEM supplemented with 10 % heat-inactivated fetal calf serum, 100 U/ml penicillin, and 100 mg/ml streptomycin. The culture medium was changed after 24 h and then every 3 days. Two weeks later, microglia were obtained by intensive washing and shaking the culture (250 rpm) for 1 h at 37 °C. The purity of the microglia was >97 % as determined by flow cytometry to measure the percentage of CD11b^+^ cells.

### Quantitative RT-PCR

Real-time quantitative RT-PCR (qPCR) was performed as described using gene-specific primer sets (Table 1). Gene expression was assessed in triplicate and normalized to a reference gene, *Actb*.

**Table 1:** Gene-specific primers used for real-time PCR assays

Gene	Forward primer	Reverse primer
*Il1b*	AAGCCTCGTGCTGTCGGACC	TGAGGCCCAAGGCCACAGGT
*Il6*	CACAGAGGATACCACTCCCAACA	TCCACGATTTCCCAGAGAACA
*Tnf*	CATCTTCTCAAAATTCGAGTGACAA	CCAGCTGCTCCTCCACTTG
*Ifna*	TGACCTCAAAGCCTGTGTGATG	AAGTATTTCCTCACAGCCAGCAG
*Ifnb*	AGCTCCAAGAAAGGACGAACAT	GCCCTGTAGGTGAGGTTGATCT
*Peli1*	CCTTGTCCATGTAAGTTTCTC	CAGAGTTCAGAAGTCTGGAACT
*Actb*	CGTGAAAAGATGACCCAGATCA	CGTGAAAAGATGACCCAGATCA

### Immunoblot (IB)

Primary microglia were stimulated with 100 ng/ml LPS for the indicated time period and lysed in RIPA buffer. The whole cell extract were separated via 8.25 % SDS-PAGE, transferred to PVDF membranes, blocked and subject to IB analysis.

### Statistical analysis

One-way ANOVA, where applicable, was performed to determine whether an overall statistically significant change existed before the Student’s *t*-test to analyze the difference between any two groups. Data are presented as means ± SD. A *P*-value less than 0.05 is considered statistically significant.

## Abbreviations

IFN-I: Type I interferon
CNS: Central nervous system
TLR: Toll-like receptor
VSV: Vascular stomatitis virus
IFNAR: Type I IFN receptor
PRR: Pattern-recognition receptor
RLR: RIG-I-like receptor
IRAK: IL-1 receptor-associated kinase
MEF: Mouse embryonic fibroblast
WT: Wild-type
KO: Knockout
GFP: Green fluorescence protein
CSF: Cerebrospinal fluid
OB: Olfactory bulb
